# Unveiling the anti-neoplastic potential of *Schistosoma mansoni*-derived antigen against breast cancer: a pre-clinical study

**DOI:** 10.1186/s40001-025-02531-5

**Published:** 2025-04-17

**Authors:** Maha Mohamed Eissa, Sonia Rifaat Ahmed Allam, Cherine Adel Ismail, Rasha Abdelmawla Ghazala, Nahla El Skhawy, Inass Ibrahim Ahmed Zaki, Eman Ibrahim El-said Ibrahim

**Affiliations:** 1 Department of Medical Parasitology, Faculty of Medicine, Alexandria University, Al-Moassat Medical Campus, Alexandria, Egypt; 2 Department of Clinical Pharmacology, Faculty of Medicine, Alexandria University, Alexandria, Egypt; 3 Department of Medical Biochemistry, Faculty of Medicine, Alexandria University, Alexandria, Egypt; 4 Department of Pathology, Faculty of Medicine, Alexandria University, Alexandria, Egypt

**Keywords:** Parasite immunotherapy, *Schistosoma*, MCF-7, Breast cancer

## Abstract

**Background:**

Cancer is a global health concern, with millions of new cases and deaths annually. Recently, immunotherapy has strengthened cancer treatment by harnessing the body's immune system to fight cancer. The search for advanced cancer immunotherapies has expanded to explore pathogens like parasites for their potential anti-neoplastic effects. While some parasites have shown promising results, the role of *Schistosoma mansoni* in breast cancer remains unexplored.

**Methods:**

This pre-clinical study investigated the anti-neoplastic potential of autoclaved *Schistosoma mansoni* antigen against breast cancer. In vitro, autoclaved *Schistosoma mansoni* antigen was evaluated on the MCF-7 human breast cancer cell line, while in vivo experiments used a chemically induced breast cancer rat model to evaluate tumour growth, liver enzyme levels, and immune response. Histopathological and immunohistochemical analyses assessed changes in tumour tissue, cell proliferation (Ki-67), angiogenesis (CD31), immune cell infiltration (CD8^+^ T cells), regulatory T cells (FoxP3^+^), and programmed death ligand 1 (PD-L1) expression.

**Results:**

In vitro, autoclaved *Schistosoma mansoni* antigen significantly reduced MCF-7 cell viability in a dose- and time-dependent manner. In vivo, autoclaved *Schistosoma mansoni* antigen treatment significantly reduced tumour weight and volume, improved liver enzyme levels, increased tumour necrosis, and decreased fibrosis. Immunohistochemical analysis revealed decreased Ki-67 and CD31 expression, indicating reduced cell proliferation and angiogenesis, respectively. Autoclaved *Schistosoma mansoni* antigen also enhanced immune responses by increasing CD8^+^ T cells infiltration and decreasing FoxP3^+^ expression, resulting in a higher CD8^+^ T cells/FoxP3^+^ ratio within the tumour microenvironment. Notably, PD-L1 expression was also downregulated, suggesting potential immune checkpoint inhibition.

**Conclusions:**

Autoclaved *Schistosoma mansoni* antigen demonstrated potent anti-neoplastic activity, significantly reducing tumour growth and modulating the immune response within the tumour microenvironment. These results highlight autoclaved *Schistosoma mansoni* antigen's potential as a novel immunotherapy for breast cancer.

## Background

Cancer is a serious public health challenge and the second leading cause of death worldwide. It is estimated that by 2040, there will be 28 million new cases and 16.2 million deaths globally [1]. Current cancer treatment options include surgery, chemotherapy, radiotherapy, and hormonal therapy with newer therapies such as immunotherapy, targeted therapy, personalized medicines, and gene therapy [2]. Despite advancements in cancer treatment, global cancer rates are rising. Additionally, resistance to current cancer therapies is a significant challenge, highlighting the importance of ongoing research to develop more effective and innovative treatments [3].

Cancer immunotherapy has emerged as a forefront anti-tumour strategy, creating an indelible mark in cancer therapeutics [4]. An up-growing list of immunotherapeutic agents is clinically used, including adoptive T cell therapy, vaccines, and immune checkpoint blockade, among others [5]. Although it sounds genius to utilize cancer-derived antigens as immunotherapeutic tools, the results have been unsatisfactory [6]. Therefore, exploiting unfamiliar, highly immunogenic antigens from a non-host origin can be a detour to overcome such obstacles.

Pathogens are among the waiting members in the queue relying on their immunomodulatory role. This has been strongly supported by the hygiene hypothesis, which speculates the occurrence of autoimmune diseases and cancer as a consequence of limited exposure to infectious agents [7]. Strong evidence documented that co-infection with enteric parasites such as *Hymenolepis nana*, *Schistosoma mansoni* (*S. mansoni*), and *Trichuris trichiura* was associated with a reduced risk of severe COVID- 19 occurrence in African patients [8]. Despite the promising immunomodulatory role of live parasites in immune diseases, their use is quite a challenge, especially in already immunosuppressed cancer patients. Attenuated parasites could be an alternative; however, there is a need to explore other approaches such as utilizing parasitic-derived molecules or antigens that could provide a safer and more acceptable option for human use [9]. Autoclaved parasitic antigens enclose all the essential parasitic components. They are potent immunostimulants, inducing significant homologous protective immunity against experimental schistosomiasis [10], trichinellosis [11], toxoplasmosis [12], and leishmaniasis [13]. Additionally, they are easy to prepare, safe, and stable for long-term storage [14]. Moreover, they have shown promise in reducing adjuvant-induced arthritis [15], psoriasis [16], and cancer [17–19] in animal models.

Breast cancer constitutes a continuous alarming threat, with about 2.3 million women being diagnosed with this disease and contributing to 670,000 deaths annually, according to WHO statistics in 2024 [20]. Some helminth and protozoan parasites, such as *Echinococcus granulosus* (*E. granulosus*) [21–26], *Taenia solium* (*T. solium*) [27], *Trichinella spiralis* (*T. spiralis*) [28], *Plasmodium* (*P.*) species (spp.) [29], *Toxoplasma gondii* (*T. gondii*) [18], *Besnoitia jellisoni* (*B. jellisoni*) [30], *Neospora caninum* [31], *Leishmania* (*L.*) strains [32], and *Trypanosoma cruzi* (*T. cruzi*) [33] have induced promising anti-cancer potency against breast cancer in both in vitro and in vivo experiments.

Schistosomiasis is a neglected tropical parasitic disease that affects around 250 million people in tropical and subtropical regions worldwide [34]. It is caused by the *Schistosoma* spp., which can survive within the host for up to 30 years. While the association between *Schistosoma haematobium* and carcinogenesis is well-documented [35], the link between *S. japonicum* and hepatocellular carcinoma, as well as *S. mansoni* and colorectal cancer, remains unclear and is a subject of debate [36, 37]. The parasite releases antigens with immunomodulatory properties at different developmental stages, leading to immune modulation [38]. This finding has raised interest in applying *Schistosoma*-derived antigens as immunotherapeutic potential in the treatment of many diseases such as diabetes [39], allergic airway inflammation [40], chemically induced colitis [41, 42], arthritis [15], and cancers [17, 43–46]. Parasite-based immune therapy has shown promising anti-neoplastic potency against various experimental cancer models, including colon [46, 47], liver [48], lung [49], and breast [29] cancers, among others.

Interestingly, several experimental studies have demonstrated the anti-neoplastic activity of *S. mansoni* against colon cancer [17, 46], sarcoma [43], and histiocytoma [50] as well as *S. japonicum* against hepatocellular carcinoma [45] and leukaemia [44]*.*

Of particular concern, *S. mansoni* and its larval stage have been found to express human cancer-associated antigens, N-acetylgalactosamine O-serine/threonine (Tn) and Thomsen Friedenreich (TF) antigens, like the dog tapeworm, *E. granulosus.* The later parasite has demonstrated potent anti-neoplastic activity against various cancers, including breast cancer [22, 24, 25, 51]. Such findings raise interest in *S. mansoni* for its potential anti-neoplastic activity, particularly in breast cancer, an unexplored topic that warrants investigation.

In this view, the present study was designed to evaluate the anti-neoplastic potential of autoclaved *Schistosoma mansoni* antigen (A*Sm*A) against breast cancer, both in vitro using the MCF-7 human breast cancer cell line and in vivo using a chemically induced breast cancer rat model.

## Material and methods

### Experimental animals

Experimental animals (mice and rats) were obtained from the Department of Medical Parasitology, Faculty of Medicine, Alexandria University. Animals were housed in appropriate cages under standard laboratory conditions (27 ± 2 °C; 70–80% humidity; 12-h light/dark cycle) with a standard pellet diet and water ad libitum.

### *S. mansoni* maintenance and antigen preparation

Maintenance of *S. mansoni* life cycle was conducted between *Biomphalaria alexandrina* snails and male Swiss strain albino mice at the Department of Medical Parasitology, Faculty of Medicine, Alexandria University. For the preparation of A*Sm*A, the freshly collected cercariae were transferred into screw-capped vials and autoclaved at 121 °C, under a pressure of 15 lb for 15 min [15]. The vials were kept at − 20 °C until being lyophilized for later use. The protein content of A*Sm*A was quantified using a NanoDropTM 2,000 spectrophotometer and expressed in mg/ml.

### Evaluation of the anti-neoplastic effect of A*Sm*A

1. **In** **vitro** **experimental** **study**

#### Chemicals and reagents

Dulbecco's modified Eagle medium (DMEM), dimethyl sulfoxide (DMSO), 3-[4,5-dimethylthiazol- 2-yl]− 2,5-diphenyltetrazolium bromide (MTT), and 10% foetal bovine serum were purchased from Sigma-Aldrich. Antibiotics (streptomycin and amphotericin-B), L-glutamine, and sodium pyruvate were purchased from Thermo Fisher Scientific. Cisplatin was purchased from Sun Oncology Pharmacy.

#### Maintenance of MCF-7 human breast cancer cell line

MCF-7 cells were maintained at the Center of Excellence for Research in Regenerative Medicine and its Applications, Faculty of Medicine, Alexandria University, Egypt. Cells were cultured and maintained in a sterile 24-well plate in complete DMEM, supplemented with 10% fetal bovine serum, 2 mM L-glutamine, 1 mM sodium pyruvate, streptomycin (100 mg/ml), and amphotericin-B (5 mg/ml). The plate was incubated under a humidified atmosphere of 5% CO_2_ at 37 °C in an automated CO_2_ incubator until confluent.

#### Assessment of A*Sm*A cytotoxicity on MCF-7 cells

The cytotoxicity of A*Sm*A on MCF-7 cells was evaluated using MTT assay as per manufacturer instructions. Briefly, MCF-7 breast cancer cells were seeded in a clear, flat-bottom 96-well plate at a density of 8,000 cells per well in 100 µl of growth medium and incubated overnight for 24 h. A*Sm*A was serially diluted with growth media to prepare different concentrations (5, 10, 20, 30, and 40 μg/ml). Cisplatin (5, 10, 20, 30, and 40 μg/ml) was included as a positive therapeutic control. For each concentration and time course study, negative control samples were included that remained untreated and received an equal volume of the culture media. All experiments were conducted for 24 and 48 h. After the 24- and 48-h incubation periods 10 µl of MTT labelling reagent was added to each well and incubated at 37.5 °C for 4 h in a humidified 5% CO_2_ atmosphere. One hundred μl DMSO was added to dissolve the formazan crystals. Finally, optical density was measured at 570 nm using a microplate reader (Benchmark, BIO-RAD). All experiments were performed in triplicate. To calculate the cell viability percentage, the mean absorbance of each concentration was divided by the mean absorbance of the negative control and multiplied by 100.

2. **In vivo** **experimental** **study**

#### Chemicals and reagents

Dimethylbenz[a]-anthracene (DMBA) was purchased from Sigma-Aldrich. Primary antibodies for immunohistochemical staining were purchased from Roche Diagnostics; anti-Ki-67 (Cat No. 790–4286), anti-CD8^+^ T cells (Cat No. 790–2931), anti-forkhead box protein 3 (FoxpP3^+^) (Cat No. 760–4611) anti-PD-L1 (Cat No. 790–4860) and anti-CD31 (Cat No. 760–4378).

#### Induction of experimental breast cancer in rats and experimental design

The experiment was conducted on 8-week-old female Wistar rats weighing about 150–200 g. Thirty rats were included in this study; ten were randomly chosen and served as normal controls. Breast cancer was chemically induced in the remaining 20 rats by a single subcutaneous injection of 25 mg DMBA beneath the 3rd mammary gland. DMBA was emulsified in 0.5 ml physiological saline and 0.5 ml sunflower oil, per the manufacturer’s instruction. Rats were regularly checked and palpated twice a week to check for the development of palpable tumours. Any rat that developed signs of humane endpoints as reported by Silva J et al. [52] was immediately euthanized [52].

Rats with at least one tumour reaching 0.5 cm in diameter were included sequentially in the study. They were randomly and equally divided into a DMBA-induced group (DMBA group) and DMBA-induced, A*Sm*A-treated group (DMBA-A*Sm*A-treated group), in which tumour-bearing rats were treated with two doses, two weeks apart of 5 μg/kg A*Sm*A intradermally over the sternum. Rats in both the normal and non-treated tumour-bearing groups received an equal volume of saline instead. The experiment was replicated to confirm the findings. The data presented herein are the average of both experimental replicates.

#### Biochemical analysis and tumour excision

Two weeks after the second A*Sm*A dose, rats were anaesthetized with thiopental sodium (45 mg/kg, intraperitoneal), and blood was collected for biochemical analysis. The serum was separated for biochemical analysis of aspartate transaminase (AST) and alanine aminotransferase (ALT). Following, rats were euthanized by an over-dose of thiopental sodium anaesthesia (150 mg/kg, intraperitoneal). Mammary glands from all groups were carefully dissected and subjected to pathological and immunohistochemical studies.

#### Pathological analysis

Excised mammary tissues and tumour masses from all DMBA groups and DMBA- A*Sm*A-treated groups were grossly examined for assessment of consistency. Then, they were weighted and measured for tumour volume calculation using a vernier digital caliper micrometre. Tumour volume (V) was calculated as V (mm^3^) = (L × W^2^)/2, where L is tumour length and W is tumour width. Subsequently, mammary tissues were preserved in 10% formalin and processed for haematoxylin and eosin (H&E) stain and Masson trichrome stain to evaluate the histopathological changes and fibrotic status, respectively. Five high-power fields at × 200 magnification per section were randomly photographed and analysed semi-quantitatively using ImageJ software Version 1.54 m. The necrotic area percentage (necrosis index) was calculated as the necrosis area divided by the total area of the section multiplied by 100. The percentage of fibrosis area (fibrosis index) was calculated as the total area of fibrosis divided by the total area of the section multiplied by 100.

#### Immunohistochemical (IHC) staining and analysis

Five-micron-thick sections from excised mammary glands and tumour tissues were cut and mounted on positively charged slides. Sections were deparaffinized, rehydrated in xylene and descending serial alcohol solutions, and then rinsed with phosphate buffer saline. IHC was performed utilizing Envision detection system following the manufacturer’s instructions. Visualization was performed by incubation of the sections in a solution of 3,3′-diaminobenzidine. After washing, the sections were counterstained with Mayer’s haematoxylin and mounted. The following antibodies were applied separately to the mammary tissue sections from the studied groups. The morphometric analysis was performed after digital images were captured and the immunopositively stained cells were counted using ImageJ software Version 1.54 m.

1. ***Ki-67*** ***IHC*** ***analysis***

Anti-Ki-67 antibody was used to evaluate the cellular proliferation. The Ki-67 proliferative index was calculated by dividing the number of Ki-67-positive cells by the total number of counted cells in five high-power fields, multiplied by 100 at × 400 magnification per section.

2. ***CD8***^***+***^ ***T*** ***cells*** ***and*** ***FoxP3***^***+***^ ***IHC*** ***analysis***

Anti-CD8^+^ T cells and anti-FoxP3^+^ antibodies were used to evaluate the immunogenic pattern.

Immunohistochemical stains for CD8^+^ T cells and FoxP3^+^ stromal tumour-infiltrating lymphocytes were considered positive when evident membranous/cytoplasmic or nuclear immunoreactions, respectively, were noted.

The quantification of CD8^+^ T cells, and FoxP3^+^ was estimated. Four non-overlapping high-power fields at × 400 magnification per section were captured. Subsequently, the CD8^+^ T cells/FoxP3^+^ ratio was calculated.

3. ***PD-L1*** ***IHC*** ***analysis***

PD-L1 expression was scored according to a combined positive score, which was defined as the number of PD-L1-staining cells (tumour cells and mononuclear inflammatory cells) × 400 magnification divided by the total number of viable tumour cells, multiplied by 100. Any specimen with a PD-L1 cut-off ≥ 1% is considered as positive. Then, the average number of positive-stained cells in five non-overlapping high-power fields at × 400 magnification was counted per section.

4. ***CD31*** ***IHC*** ***analysis***

An anti-CD31 antibody was used to evaluate angiogenesis. The micro-vessels were counted in five randomly selected high-power fields at × 200 magnification per section. Countable micro-vessels were defined according to the criteria established by Weidner et al. [53]. The average number of micro-vessels was defined as micro-vessel density.

The validity of each procedure was verified with the staining of positive controls (breast adenocarcinoma as Ki-67-positive control, tonsil sections as CD8^+^ T cells, FoxP3^+^ and CD31-positive control, and normal human placental tissue as PD-L1-positive control). Meanwhile, negative control was used by omission of the primary antibody.

### Statistical analysis

Data were fed to the computer and analysed using the IBM SPSS software package version 23.0. Quantitative data were described using mean and standard error of the mean (SE). The significance of the obtained results was judged at P < 0.05. F-test (ANOVA) was applied for normally distributed quantitative variables to compare between more than two groups, and post hoc test (Tukey) for pairwise comparisons. The Chi-square test was applied for categorical variables, to assess the survival percentage between different groups. The data presented are the average of two experimental replicates.

## Results

### In vitro* effect of ASmA on MCF-7 breast cancer cells*

**Fig. 1:**
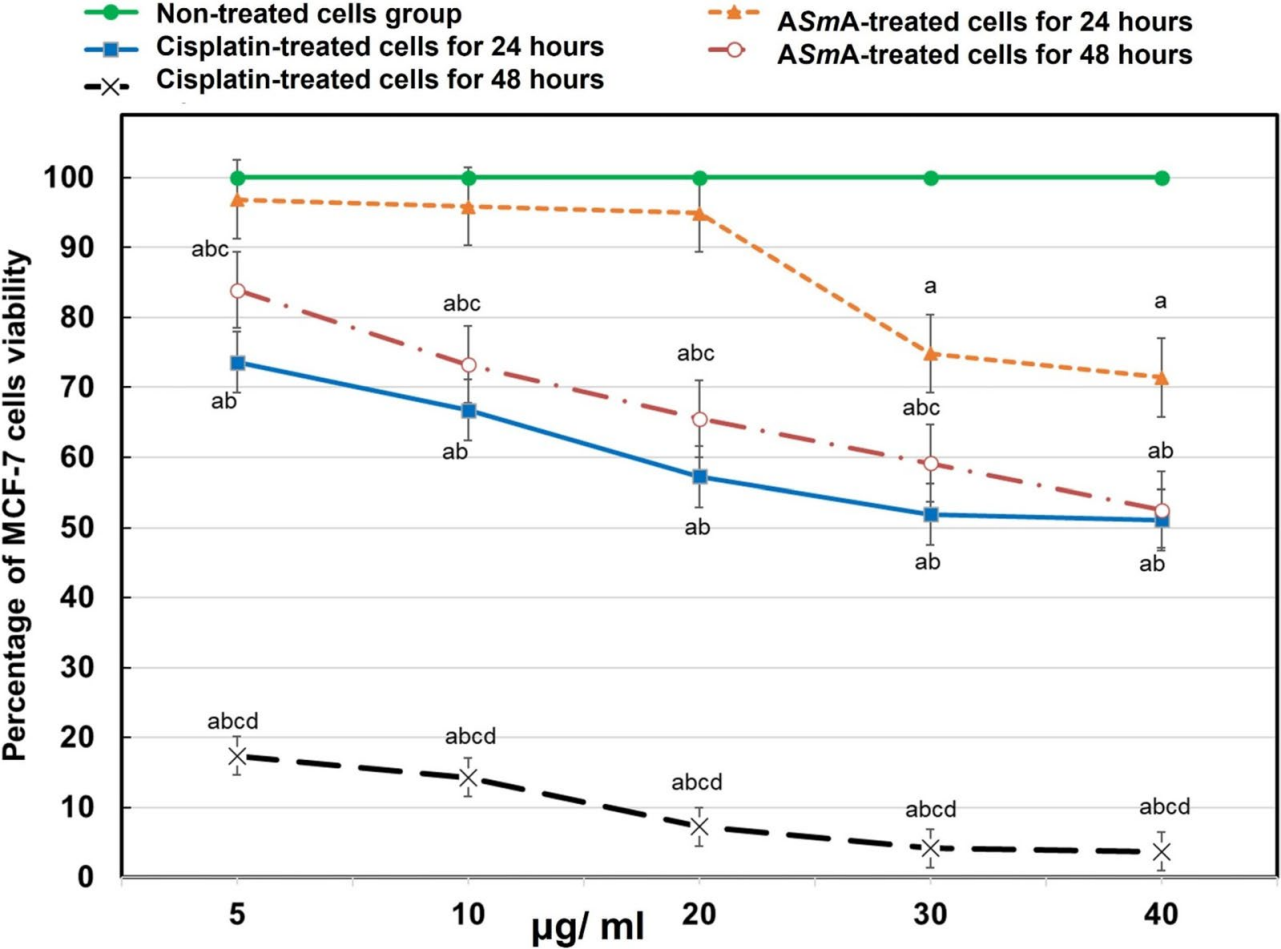
The graphical representations of percentage of MCF-7 cell viability using MTT assay after treatment with cisplatin and A*Sm*A for 24 and 48 h. Data are significant at P < 0.05. ^a^Significant versus non-treated cells. ^b^Significant versus A*Sm*A 24 h incubation. ^c^Significant versus cisplatin 24 h incubation. ^d^Significant versus A*Sm*A 48 h incubation. *ASmA* autoclaved *Schistosoma mansoni* antigen

As shown in Fig. 1, cisplatin, used as the positive control, caused a substantial reduction in cell viability at both 24 and 48 h compared to non-treated cells. On the other hand, A*Sm*A induced a reduction in cell viability of MCF-7 cells in a concentration- and time-dependent manner. After 24 h of incubation, A*Sm*A induced a significant reduction in cell viability at concentrations of 30 and 40 μg/ml, compared to non-treated cells. Meanwhile, after 48 h of incubation, all tested doses of A*Sm*A induced significant cytotoxic activity compared to non-treated cells. Interestingly, there was no statistically significant difference in the inhibition of cell viability between cisplatin at 40 μg/ml for 24 h of incubation and A*Sm*A at 40 μg/ml for 48 h of incubation.

### In vivo effect of A*Sm*A on chemically induced breast cancer rat model

#### Survival percentage

Compared with normal control rats, both DMBA and DMBA-A*Sm*A-treated groups were generally in good condition. DMBA group exhibited a survival percentage of 75% meanwhile DMBA-A*Sm*A-treated group exhibited 80% survival by the end of the experiment (Table 1).

**Table 1:** Effect of A*Sm*A treatment on survival percentage, biochemical analysis of ALT, AST, tumour volume (mm^3^) and tumour weight (in gram) in DMBA-induced mammary tumour rat model

	Normal group	DMBA group	DMBA-A*Sm*A-treated group	p
Survival percentage	100%	75%	80%	0.066
Liver function				
ALT (U/L)	22.77 ± 1.82	67.13 ± 3.56^a^	45.87 ± 2.33^ab^	< 0.001
AST (U/L)	15.27 ± 1.58	39.06 ± 2.24^a^	25.77 ± 1.95^ab^	< 0.001
Volume (× 10^3^) mm^3^	0.0 ± 0.0	5.96 ± 1.41^a^	1.40 ± 0.35^ab^	< 0.001
Weight in gramme	0.0 ± 0.0	6.08 ± 1.24^a^	2.40 ± 0.31^ab^	< 0.001

Values were expressed as mean ± SE. Data are significant at P < 0.05

^a^Significant versus control group

^b^Significant versus DMBA group

DMBA dimethylbenz[a]-anthracene, *ASmA* autoclaved *Schistosoma mansoni* antigen, ALT alanine aminotransferase, AST aspartate aminotransferase, SE standard error

### Biochemical analysis

Chemical induction of breast cancer in female rats using DMBA has significantly elevated liver enzymes (ALT and AST) compared to the normal group. However, in DMBA-A*Sm*A-treated group, A*Sm*A has significantly reduced the elevated ALT and AST compared to the DMBA group, as shown in Table 1.

### Gross examination

**Fig. 2:**
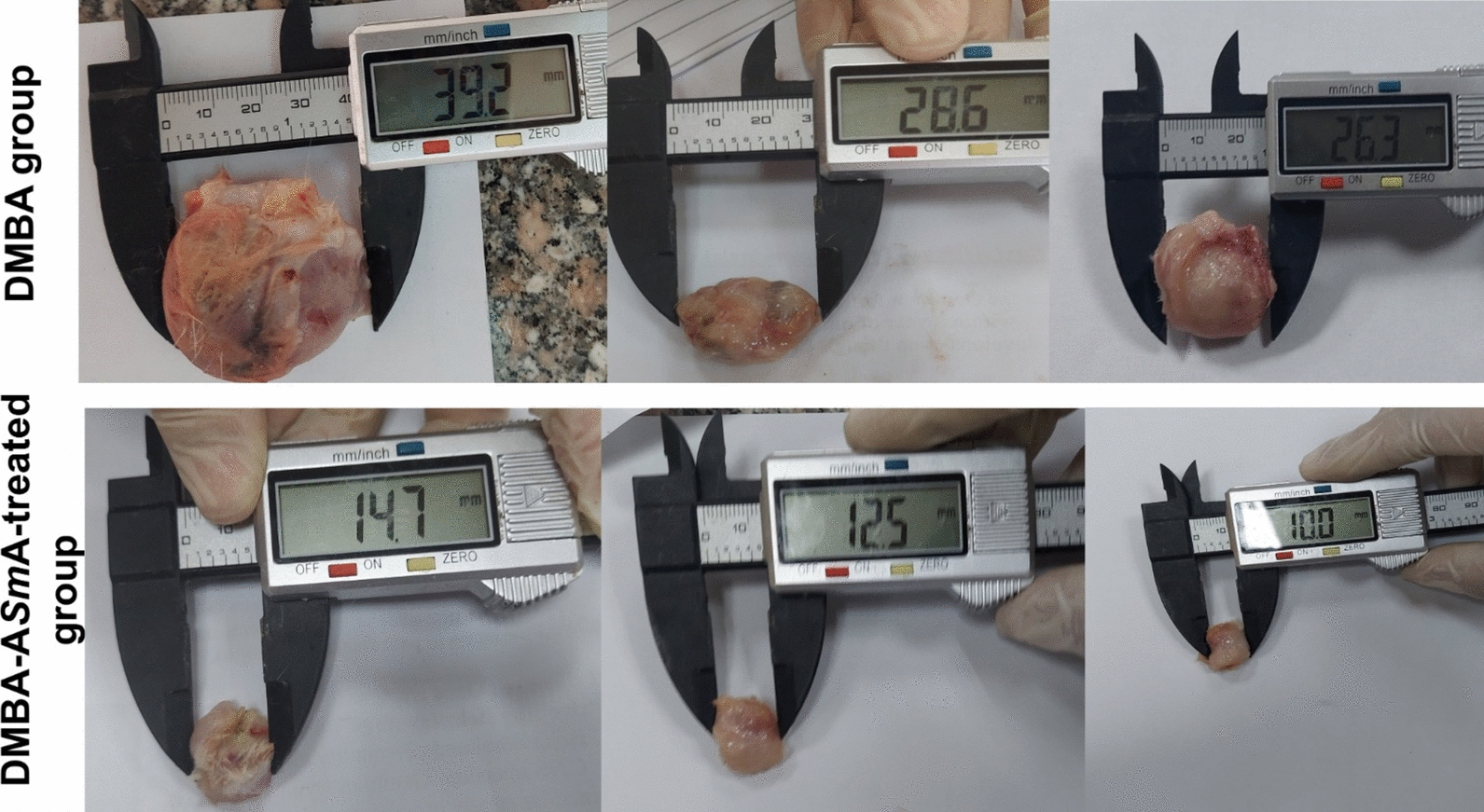
Representative gross photographs of tumour tissues excised from the untreated DMBA group and DMBA- A*Sm*A-treated group. DMBA dimethylbenz[a]-anthracene, *ASmA* autoclaved *Schistosoma mansoni* antigen

Subcutaneous masses excised from mammary sites from all DMBA and DMBA-A*Sm*A-treated groups were grossly examined. The DMBA-A*Sm*A-treated group showed a significant reduction in weights and volume compared to the DMBA group, as shown in Table 1. Additionally, the tumour masses were less firm in consistency compared to the DMBA group. A photographic image of samples of excised mammary tissues is seen in Fig. 2.

### Histopathological examination

**Fig. 3:**
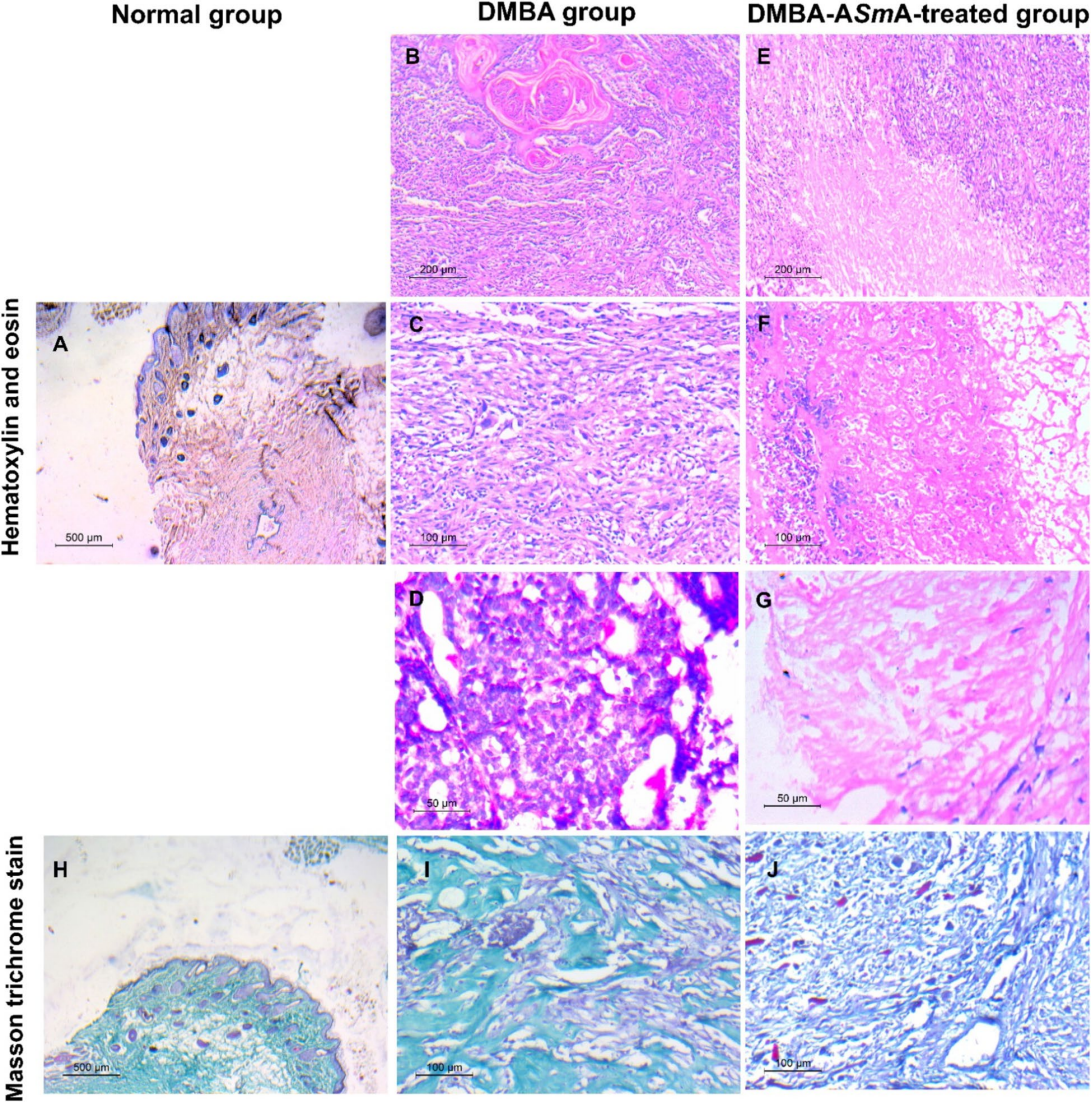
Microphotograph of rat mammary tissue stained with haematoxylin and eosin (H&E) (**A**–**G**) and Masson trichrome (**H–J**). Section of control rat mammary tissue showing normal mammary architecture (**A** in H&E stain and **H** in Masson trichrome) × 40. **B**–**D and I** Mammary tissue section of DMBA group showing invasive ductal carcinoma with squamous metaplasia grade III **B** × 100, higher magnification showed atypical mitosis and bizzar shaped malignant cells with hyperchromatic nuclei **C** × 200 & **D** × 400; and marked dense fibrosis **I** × 200. **E**–**G and J** Mammary tissue section of DMBA-A*Sm*A-treated group showing extensive areas of necrosis and inflammatory cells infiltrations with foci of remnants invasive carcinoma **E** × 100, **F** × 200 and **G** × 400 and less dense fibrosis **J** × 200. DMBA: dimethylbenz[a]-anthracene; A*Sm*A: autoclaved *Schistosoma mansoni* antigen

**Fig. 4:**
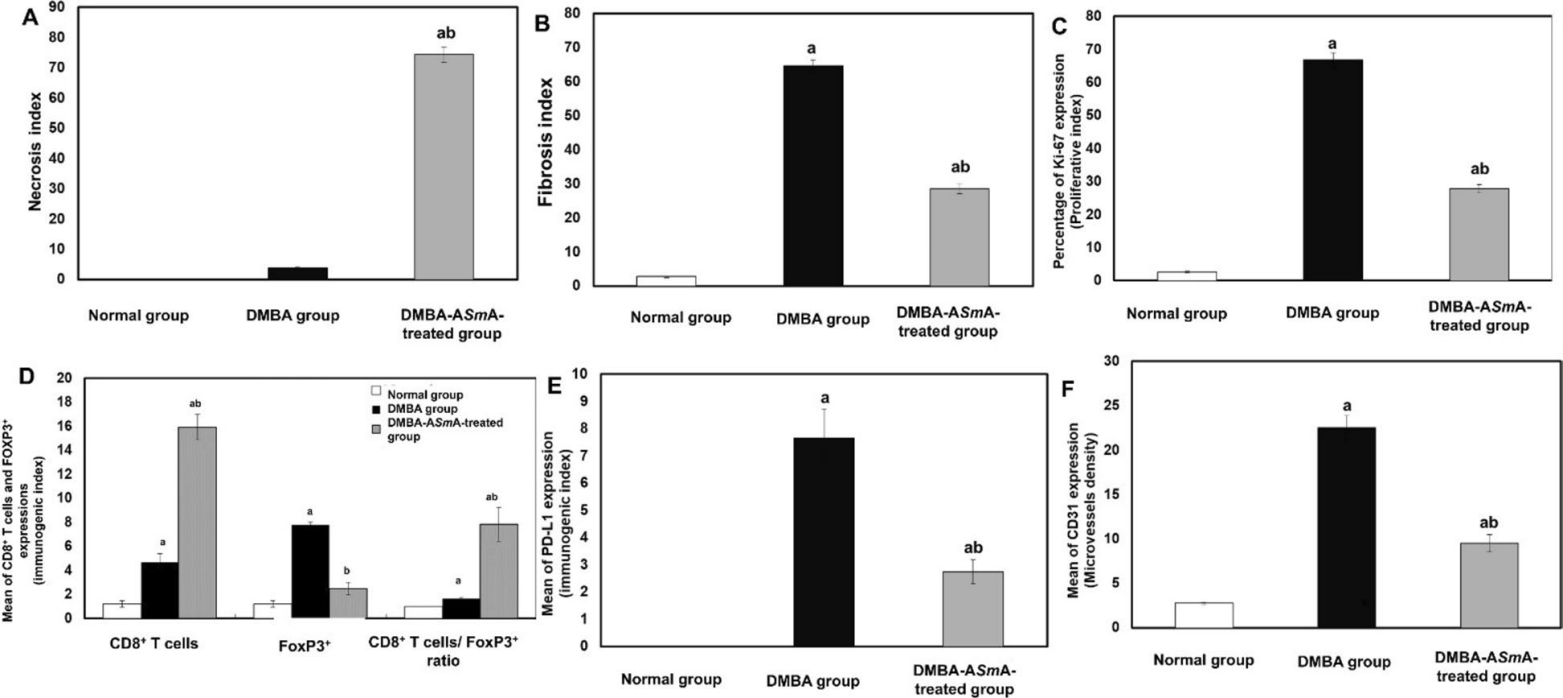
Morphometric analysis of: **A** necrosis index, **B** fibrosis index, **C** percentage of Ki-67 expression (proliferation index), **D** CD8^+^ T cells, FoxP3^+^ cells and CD8^+^ cells/FoxP3^+^ ratio, **E** PD-L1 expression, and **F** CD31 micro-vessels density Values were expressed as mean ± SE. Data are significant at P < 0.05. ^a^Significant versus normal control group ^b^Significant versus DMBA group. DMBA dimethylbenz[a]-anthracene, *ASmA* autoclaved *Schistosoma mansoni* antigen, SE standard error

Histological staining of tissue sections from mammary tissues of the normal group by H&E showed pathologically unremarkable breast tissue (Fig. 3A). Meanwhile, mammary tumour sections from the DMBA group revealed invasive ductal carcinoma with squamous metaplasia (Fig. 3B). At a higher power magnification, they showed atypical mitosis and bizarre-shaped malignant cells with hyperchromatic nuclei (Fig. 3C and D). Sections of mammary tumours from DMBA-A*Sm*A-treated group showed extensive areas of necrosis and inflammatory cell infiltrations with foci of remnants of invasive carcinoma (Fig. 3E, F & G) with a statistically significant increase in necrosis index in the DMBA-A*Sm*A-treated group compared to the DMBA group (Fig. 4A).

Masson trichrome stain sections from normal breast tissues showed collagenous fibres that were depicted in a whorl pattern around normal epithelial cells (Fig. 3H). Meanwhile, a section from DMBA group showed marked dense fibrosis surrounding the tumour with intraductal fibrosis (Fig. 3I). On the other hand, DMBA-A*Sm*A-treated group showed less dense fibrosis (Fig. 3J) with a statistically significant reduction in fibrosis index compared to the DMBA group (Fig. 4B).

### Immunohistochemical examination

**Fig. 5:**
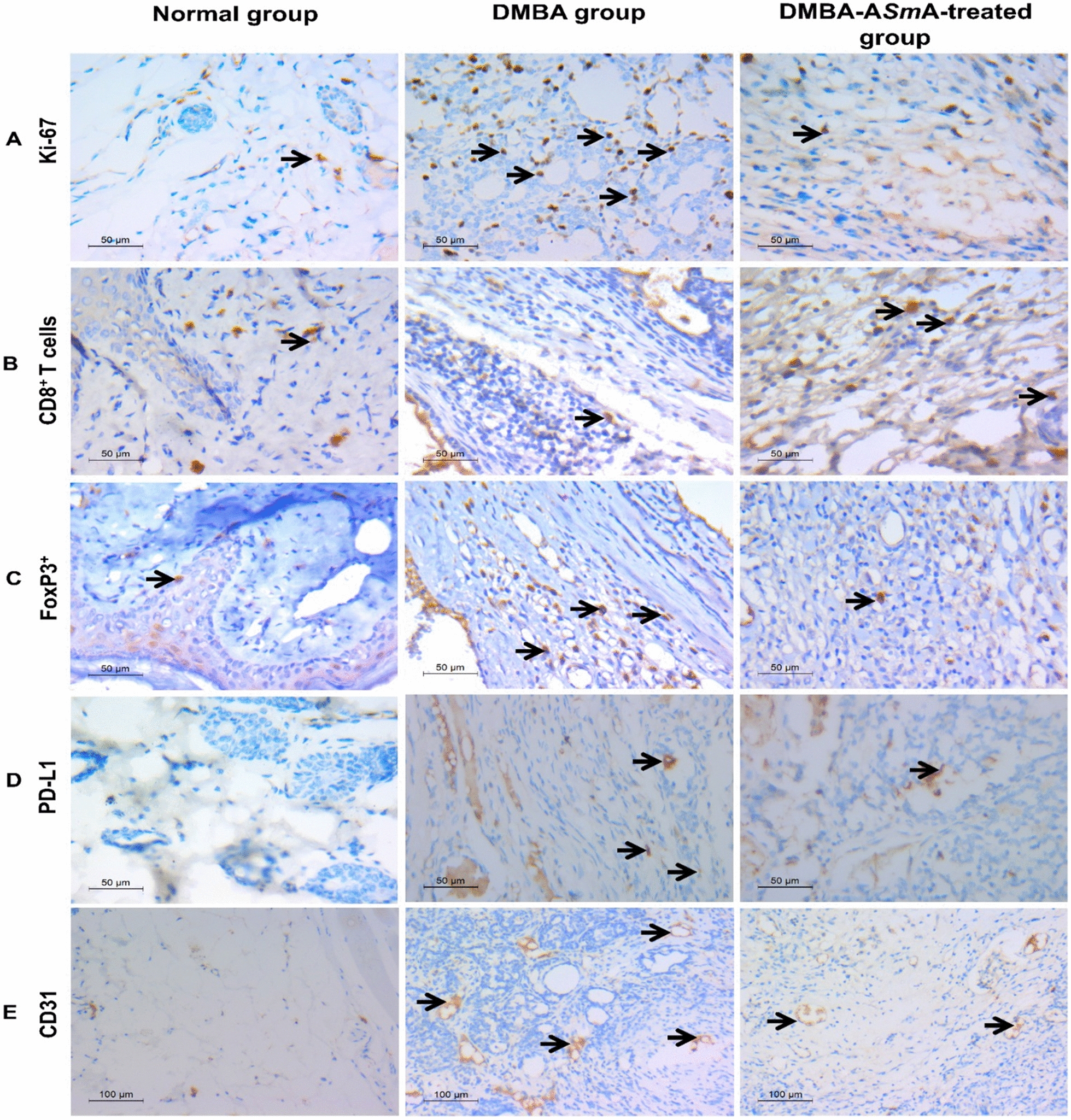
Microphotograph of rat mammary tissue stained with immunohistochemical analysis. **A** Ki-67 expression showing low proliferative index in normal group, high proliferative index in DMBA group, reduction in proliferative index in DMBA-A*Sm*A-treated group. **B** CD8^+^ T cells expression in cell membrane of infiltrating T cells in mammary tissue sections. CD8^+^ antibody expression was reduced in DMBA group and were elevated in DMBA-A*Sm*A-treated group. **C** FoxP3^+^ expression in the perinuclear and nuclear pattern of infiltrating T cells in mammary tissue sections. FoxP3.^+^ expression was increased in DMBA group and reduced in DMBA- A*Sm*A-treated group. **D** PD-L1 expression, it was negative in sections of normal breast tissue. DMBA group showed high PD-L1 expression and DMBA-A*Sm*A-treated group showed reduction in PD-L1 expression. **E** CD31 expressions highlighting the vessels and micro-vessels, normal group exhibited a normal pattern of CD31 expression, DMBA group showed obviously nested CD31 expression in between cancer cells. DMBA-A*Sm*A-treated group showed few expressions of CD31. Black arrows point out the positive-stained cells. DMBA dimethylbenz[a]-anthracene, *ASmA* autoclaved *Schistosoma mansoni* antigen, PD-L1 programmed death ligand 1

Results of the IHC examination of Ki-67 expression in mammary tissue sections of the normal group showed scanty staining of some nuclei in the cells surrounding the ducts with a low proliferative index (Fig. 5A). Notably, sections of the mammary tissue excised from the DMBA group revealed intense staining of many nuclei representing a high proliferative index. On the other hand, in DMBA-A*Sm*A-treated breast tumour, few nuclei stained with a low proliferative index were observed. The morphometry results showed that A*Sm*A treatment induced a statistically significant reduction in proliferative index Ki-67 expression versus the DMBA group (Fig. 4C).

Regarding IHC staining of mammary tissues with the CD8^+^ T cell marker, it was stained in the cell membrane of infiltrating T cells in mammary tissue sections (Fig. 5B). CD8^+^ T cells were significantly reduced in the DMBA group compared to the normal control. However, treatment with A*Sm*A in the breast cancer model showed a strikingly significant increase in CD8^+^ T cells infiltrating the tumour compared to the DMBA group (Fig. 4D).

As regards FoxP3^+^ as a marker for T regulatory (Treg) cells, the perinuclear and nuclear pattern of FoxP3^+^ immuno-expression on tumour-infiltrating T cells was noted (Fig. 5C). It was significantly elevated in the DMBA group compared to the normal group. Treatment with A*Sm*A in the breast cancer model induced a statistically significant reduction in FoxP3^+^ cells compared to the DMBA group (Fig. 4D). These results were reflected in the ratio between CD8^+^ T cells/FoxP3^+^ that was significantly higher in the DMBA-A*Sm*A-treated group compared to the DMBA group (Fig. 4D).

As regards PD-L1 expression, it was negative in sections of normal breast tissue. The DMBA group showed expression of PD-L1 with a score ˃ 1. Thus, this model is considered PD-L1 positive (Fig. 5D). Meanwhile, A*Sm*A treatment induced a reduction in PD-L1 expression in sections from mammary tumours of the DMBA-A*Sm*A-treated group compared to the DMBA group (Fig. 4E).

Concerning IHC staining of mammary tissues with CD31 marker, it highlighted the vessels and micro-vessels in all examined mammary sections (Fig. 5E). In the normal group, the micro-vessels exhibited a normal pattern of CD31 expression. In the DMBA group, CD31 expression was obviously nested in between cancer cells. However, the DMBA-A*Sm*A-treated group showed few expressions of CD31. Calculation of the micro-vessel density revealed that the DMBA group showed a statistically significant elevation in micro-vessel density compared to the normal group, yet this was significantly dropped upon treatment with A*Sm*A versus the DMBA group (Fig. 4F).

## Discussion

A wide range of pathogens have demonstrated immunomodulatory potential in various experimental cancer models. However, only a few have achieved clinical excellence. Notable examples include Bacillus Calmette–Guérin (BCG), which has been used as an immunotherapeutic agent for urinary bladder cancer and metastatic melanoma [54, 55], and T-VEC (Imlygic^®^), a modified herpes simplex virus approved by the Food and Drug Administration (FDA) for melanoma treatment [56]. Additionally, preventive vaccines like the human papillomavirus vaccine, which reduces the risk of cervical cancer [57], and the hepatitis B virus vaccine, which prevents hepatocellular carcinoma (HCC), have also shown clinical impact [58].

Parasites are on track, and parasite-based immunotherapy has shown promising anti-neoplastic effects against various experimental cancer models [59]. Both helminths and protozoan parasites have exhibited significant anti-neoplastic activity against breast cancer cells in in vitro and in vivo animal studies [25, 29, 32]. However, the efficacy of *S. mansoni-*derived antigen as a therapeutic strategy against breast cancer remains unexplored. Therefore, it is crucial to investigate the anti-neoplastic potential of A*Sm*A against breast cancer both in vitro and in vivo to evaluate its effectiveness against breast cancer.

In the present study, MTT assay results demonstrated that A*Sm*A induced promising cytotoxic effects on MCF-7 cells in a dose- and time-dependent manner. The highest cytotoxic effect was observed after 48 h of treatment with 40 μg/ml of A*Sm*A. Notably, this cytotoxic activity was comparable to cisplatin, a clinically approved chemotherapeutic agent, at a concentration of 40 μg/ml for 24 h, commonly used in the treatment of various cancers, including breast cancer [60]. Our results are in agreement with Pekkle et al., [46] who reported that *S. mansoni* soluble egg antigen inhibited the growth of human colorectal cancer cell lines (HCT- 116 and DLD- 1) [46]. Similarly, anti-neoplastic activity was reported for *S. japonicum-*derived molecules. Specific microRNA mimics from *S. japonicum*, including *Sja-*miRNA- 7- 5p, *Sja*-miR- 3096, *Sja*-miR- 61, and *Sja*-miR- 71, have been shown to induce significant cell cycle arrest, inhibit cell proliferation, and suppress growth and migration in liver cancer cell lines such as Hepa1- 6 (mouse hepatoma), HepG2 (human hepatoma), and SMMC-7721 (human HCC) [45, 48, 61, 62]. Additionally, recombinant *Sj*16 (r*Sj*16) demonstrated significant dose- and time-dependent induction of apoptosis and cell cycle arrest in the murine myelomonocytic leukaemia cell line WEHI- 3B JCS [44].

Other helminthic parasites exhibit comparable cytotoxic effects; for instance, the recombinant *E. granulosus* Kunitz-type protease inhibitor (*Eg*KI- 1) has shown cytotoxic activity against the MCF-7 breast cancer cell line [23]. Additionally, hydatid cyst fluid antigens have been reported to exert cytotoxic effects against both the MDA-MB-231 and 4 T1 mouse breast cancer cell lines [21, 22], while calreticulin derived from *T. solium* has demonstrated similar effects on the MCF-7 cell line [27]. Moreover, studies on protozoan parasites have also highlighted notable cytotoxic activities. For instance, recombinant VAR2 CSA from *Plasmodium*, when used as a carrier for the anti-cancer drug, VDC886 (hemiasterlin analogue (KT886) conjugated with recombinant VAR2 CSA) induced strong cytotoxicity towards breast cancer cell lines MDA-MB-231 and 4 T1 [63]. Additionally, recombinant *T. cruzi* lysate and recombinant *T. cruzi* P21 have shown significant cytotoxic effects against the MCF-7 cell line [64] and MDA-MB-231 cancer cells [65], respectively.

Live parasites have also demonstrated promising anti-proliferative activities against various breast cancer cell lines. Notably, *T. gondii* tachyzoites have been shown to affect MCF-7 cells [66–68], MDA-MB-231 cells [68], and Her2/Neu-expressing mammary cancer cells [69]. Furthermore, *T. cruzi* infection impaired triple-negative breast cancer cells migration [70]. *Neospora caninum* tachyzoites were able to invade MCF-7 cells [31]. These inhibitory effects may be attributed to the invasion, replication, and aggregation of the live parasites within tumour cells, leading to their lysis.

In the present in vivo experiment, DMBA was used to induce breast cancer in a rat model. DMBA is a well-established carcinogen exhibiting immunosuppressive properties, and it induces mammary carcinomas that closely mimic human breast cancer. This is achieved by creating an immunosuppressed tumour microenvironment (TME) via diminishing tumour-inhibitory cytotoxic immune response and promoting the expansion of Treg cells. This oncogenesis is achieved through DNA damage, DNA methylation, and various genetic abnormalities [71]. Liver enzymes are widely used as pathophysiological biomarkers to monitor cancerous conditions, serving as indicators of tissue damage, tumour progression, metastasis, and drug-induced hepatotoxicity [72].

In the present study, DMBA-induced carcinogenesis was associated with a significant elevation of liver enzyme levels compared to normal control, consistent with findings from previous studies [73]. The administration of two doses of A*Sm*A exhibited a significant ameliorative effect on liver enzymes, which can be either justified by the reduction of tumour growth or restraining DMBA toxicity. These findings are in agreement with other studies that reported improved liver functions following prophylactic and therapeutic administration of the autoclaved *T. gondii* vaccine against Ehrlich solid carcinoma in the murine model [18, 19]. These also highlight the safety profile of A*Sm*A, aligning with earlier work of Eissa et al. [14], who demonstrated the absence of both local and systemic side effects, alongside normal haematological parameters and liver and kidney function tests following A*Sm*A administration [14].

Indeed, A*Sm*A administration led to a significant reduction in both tumour weight and volume compared to the DMBA group, underscoring its potential as an anti-neoplastic agent. These findings agree with previous studies demonstrating the anti-neoplastic activity of *S. mansoni* antigens in various cancer models, as well as the anti-neoplastic activity of other parasites against breast cancer in murine models. As regards *Schistosoma* parasite, an early research study, dating back to 1991, first reported the anti-neoplastic activity of *S. mansoni* antigen in a murine cancer model [50]. This is further supported by findings by Eissa et al*.* [17], which revealed the protective effects of A*Sm*A against chemically induced colon carcinogenesis in a murine model. Their study demonstrated a significant reduction in both lesion size and the number of neoplasms per mouse, alongside the activation of an immunomodulatory response [17]. Recently, Pekkle et al. [46] reported that *S. mansoni* soluble egg antigen showed inhibition of colon cancer growth in a mouse xenograft model [46]. Similarly, multiple *S. japonicum*-derived molecules have demonstrated in vivo anti-tumour properties. In a xenograft liver cancer animal model, mice inoculated with cells transfected with *Sja*-miRNA-7-5p, *Sja*-miR-3096, *Sja*-miR-61, or *Sja*-miR-71a mimics inhibited tumour growth and experienced significant reductions in tumour volume and weight. These effects were achieved through the modulation of tumour-related genes. For instance, *Sja*-miRNA-7-5p downregulated the S-phase kinase-associated protein 2 gene [45], *Sja-*miR-3096 targeted phosphoinositide 3-kinase class II alpha gene [48], *Sja*-miR-61 induced an anti-angiogenic activity by targeting the PGAM1 gene [62], and *Sja-*miR- 71a targeted the FZD4 gene [61]. The suppression of tumour growth in vivo by *Sja*-miR highlights their potential as novel and promising approaches to cancer therapy.

Similar findings have been reported using different parasites and their derived molecules in in vivo breast cancer models, demonstrating a range of anti-tumour mechanisms. Experimental infection with hydatid disease [24], *T. spiralis* [74, 75], *T. cruzi* [33], *P. yoelli* [29], *T. gondii* [30], and *B. jellisoni* [30] have been shown to suppress breast cancer development in experimental models. Additionally, live-attenuated parasites, such as attenuated *L. infantum* and *L. tropica* strains [32], as well as attenuated uracil auxotroph strain of *T. gondii* [76] demonstrated similar anti-tumour activity.

Notably, the use of parasitic antigens and their derived molecules presents a safer alternative to live or attenuated parasites for cancer therapy. Hydatid cyst wall antigens [25], hydatid cyst fluid and antigen B [21, 25, 26, 51], *Eg*KI- 1 [23], *T. cruzi* epimastigote lysates [33], and recombinant *T. cruzi* calreticulin [77] have exhibited similar promising anti-cancer effects, highlighting their potential as safe therapeutic agents.

As verified by the histopathological analysis, DMBA induced invasive ductal carcinoma. Treatment with A*Sm*A induced amelioration of the mammary cancer pathology, evidenced by reduced mitotic activity, extensive inflammatory cell infiltration, a striking increase in necrosis index, and decreased fibrosis index. The increase in the necrotic index is a valuable cancer prognostic marker, denoting the end of an aggressive battle between cancer cells and immune cells following immunotherapy [78]. Similar findings have been reported in therapeutic experiments using live-attenuated *Leishmania* strains in triple-negative 4T1 breast cancer model [32] and autoclaved *T. gondii* tachyzoites in a murine Ehrlich carcinoma model, both prophylactically and therapeutically [18, 19].

Fibrosis plays a critical role in TME, which impacts cancer cells' behaviour. While multiple reports have documented that fibrosis is a sign of a tissue healing response that encapsulates cancer cells, limiting their invasion, it is associated with poor prognosis in breast cancer due to its immunosuppressive properties [79]. Fibrosis restricts immune cell infiltration, facilitates cancer cell crosstalk, and results in a poor prognosis. In addition, fibrosis promotes a pro-tumorigenic environment through Treg cells and provides a chemo-resistant niche for tumour cells [80]. In the present study, DMBA-induced breast cancer resulted in the development of firm, solid tumours characterized by extensive fibrosis. This was accompanied by poor infiltration of CD8^+^ T cells and a high number of Treg cells within the tumour microenvironment, along with elevated PD-L1 expression, contributing to an immunosuppressive state. In contrast, tumours from the DMBA-A*Sm*A-treated group exhibited a softer consistency and a lower fibrosis index, which correlated with enhanced CD8^+^ T cells infiltration and a reduction in Treg cells. This was also associated with decreased PD-L1 expression, indicating a shift towards a more immunoreactive state and suggesting a more favourable prognosis.

Cancer cells are skilled at creating intricate TME that eludes the immune system's attempts to combat them. Key immune-resistance mechanisms within the TME include the reduction of CD8^+^ T cells, recruitment of immunosuppressive cells such as Treg cells, upregulation of immune checkpoints pathways like PD- 1/PD-L1, and the promotion of tumour neovascularization [81]. In this study, we investigated these mechanisms by assessing Ki-67 as a marker of proliferation index, CD8^+^ T cells, FoxP3^+^ and PD-L1 as markers of immune activity, and CD31 as a marker of angiogenesis. Ki-67, a cell cycle nucleoprotein, is widely used as an indicator of cell proliferation, a diagnostic marker, a prognostic tool, as well as a potential therapeutic target in cancer. A higher proliferative index indicates increased cell division, signifying a more aggressive tumour [82]. Herein, results showed that A*Sm*A treatment significantly reduced Ki-67 expression and lowered the proliferation index compared to the DMBA group. This finding harmonizes with our in vitro results, where A*Sm*A treatment suppressed the proliferation of MCF-7 cell breast cancer cells. Similar reductions in Ki-67 have been observed in other breast cancer models, such as DMBA-induced mammary tumours in rats following infection with hydatid disease [24]. Additionally, *Eg*KI-1 was shown to suppress Ki-67 expression in a triple-negative breast cancer model in mice [23].

To evaluate the immunological impact of A*Sm*A treatment, IHC staining was performed on mammary tumour sections to assess CD8^+^ T cells, FoxP3^+^, and PD-L1 expression. In the breast cancer microenvironment, cytotoxic CD8^+^ T cells are the chief effector cells responsible for immunosurveillance, directly inducing tumour cell death through the release of granzymes and perforin. Additionally, CD8^+^ T cells can trigger apoptosis in target cells via the Fas/FasL pathway, which stimulates caspase 8 activation [83]. Treg cells, on the other hand, play a central role in regulating cancer immunity by suppressing anti-tumour responses. They down-regulate CD8^+^ T cells and secrete inhibitory cytokines to dampen their cytotoxic effects [83].

PD-L1, a pro-tumorigenic factor within the TME, acts as an immune checkpoint. Upon engagement, PD-L1 reduces CD8^+^ T cell activation, proliferation, and cytotoxic function while promoting Treg cells differentiation and maintaining their functions by upregulating FoxP3^+^ expression. This creates a pro-carcinogenic immune environment that fosters tumour progression. Over the past ten years, immune checkpoint blockade therapies targeting PD-1/PD-L1 pathways have emerged as promising strategies for combating cancer [84].

In the present study, A*Sm*A treatment resulted in a statistically significant increase in CD8^+^ T cells and a decrease in FoxP3^+^ Treg cells compared to the DMBA group, leading to a higher CD8^+^ T cells/FoxP3^+^ ratio within the TME. The DMBA-induced breast cancer model exhibited PD-L1 positivity, with expression in both tumour cells and tumour-infiltrating immune cells. A*Sm*A administration effectively reduced PD-L1 expression in mammary tumour sections.

The induction of an immunostimulatory TME is postulated as a key anti-neoplastic mechanism of various parasites and their antigenic derivatives across different cancer models.

Live-attenuated* L.* strains and *P. yoelii* 17XNL were shown to enhance CD8^+^ T cells infiltration in the 4T1 breast cancer model [29, 32]. Similarly, the attenuated uracil auxotroph strain of *T. gondii* inhibited tumour growth, improved survival in 4T1-breast cancer-bearing mice, and increased the secretion of interleukin-12 and interferon-γ in both serum and the TME [76]. Additionally, both the prophylactic and therapeutic use of autoclaved *T. gondii* tachyzoites induced higher infiltration of CD8^+^ T cells, reduced Treg cells, and resulted in a higher CD8^+^ T cells/Treg ratio within the Ehrlich solid carcinoma in a mouse model [18, 19]. Furthermore, *T. cruzi* antigen has been reported to elicit a comprehensive anti-tumour response involving both cellular and humoral immune components in a chemically induced breast cancer model [33]. Likewise, infection with * P. yoelii* 17XNL demonstrated anti-neoplastic efficacy in the Lewis lung cancer mouse model by modulating the TME, characterized by significantly elevated levels of granzyme B and perforin and notably reduced PD-L1 expression [49].

Neovascularization is one of the cancer hallmarks that is essential to secure sufficient blood flow and nutrient supply to the rapidly proliferating tumour cells. It also serves as a conduit for tumour cells to be shed in the circulation, facilitating metastasis [85]. Angiogenesis has been linked to the development of an immunosuppressive TME. CD31 is a specific and well-established marker for vascular differentiation and monitoring micro-vessel density in malignant tissue. Increased micro-vessel density, as indicated by CD31 expression, is associated with poor prognosis in breast cancer. Conversely, loss of CD31 expression has been linked with enhanced immunoreactivity and increased susceptibility to cytotoxic cell-mediated killing [86]. In the present study, A*Sm*A treatment significantly reduced CD31 expression and micro-vessel density compared to the DMBA group, highlighting its potent anti-angiogenic effect. This reinforces the therapeutic potential of A*Sm*A as an effective anti-neoplastic agent. Similar anti-angiogenic effects have been reported with recombinant *T. cruzi* calreticulin in the murine mammary TA3 MTXR tumour model [87] and *Sja*-miR-61 in a murine hepatic carcinoma model [62]. Additionally, other parasites have demonstrated anti-angiogenic properties in various experimental cancer models, such as genetically attenuated *Plasmodium* sporozoites [88], *T. gondii* Me49 strain infection in a lung cancer mouse model [89], *T. gondii* lysate antigen in fibrosarcoma murine model [90], and *T. gondii* infection in the B16.F10 murine melanoma model [91].

All these findings revealed the underlying mechanisms of A*Sm*A’s anti-neoplastic activity. A*Sm*A effectively reduces tumour cell proliferation and angiogenesis while enhancing the immune response by increasing the anti-tumour immunity and modulation of the TME as demonstrated by infiltration of CD8^+^ T cells, inhibition of Treg cells, and downregulating PD-L1 expression within the TME. This collective action disrupts tumour progression and establishes A*Sm*A as a promising anti-cancer therapeutic.

## Conclusions

This study provides convincing evidence of the anti-neoplastic potential of A*Sm*A against breast cancer, both in vitro and in vivo. A*Sm*A not only inhibited breast cancer cell proliferation, but also modulated key aspects of the tumour microenvironment, including angiogenesis, immune cell infiltration, and immune checkpoint regulation. These effects led to significant suppression of breast cancer tumour growth in a rat model. The results suggest that A*Sm*A could be a promising novel immunotherapeutic agent for breast cancer. Future research should focus on exploiting the multifactorial anti-neoplastic value of A*Sm*A such as investigating its potential synergistic role with other cancer immunotherapeutic agents, like PD-L1 blockade. Additionally, identification and molecular characterization of the most antigenic component of A*Sm*A are crucial steps towards exploring its clinical relevance in cancer therapy.

## Data Availability

No datasets were generated or analysed during the current study.

## Abbreviations

ALT: Alanine aminotransferase
A*Sm*A: Autoclaved *Schistosoma mansoni* antigen
AST: Aspartate transaminase
*B*. *jellisoni*: *Besnoitia* *jellisoni*
DMBA: Dimethylbenz[a]-anthracene
DMEM: Dulbecco's modified Eagle medium
DMSO: Dimethylsulfoxide
*E*. *granulosus*: *Echinococcus* *granulosus*
EgKI- 1: Echinococcus granulosus Kunitz-type protease inhibitor- 1
FDA: Food and Drug Administration
FoxP3*+*: Forkhead box protein 3
H&E: Haematoxylin and eosin
HCC: Hepatocellular carcinoma
IHC: Immunohistochemical
*L*.: *Leishmania*
MTT: 3-[4,5-Dimethylthiazol- 2-yl]- 2,5-diphenyltetrazolium bromide
PD-L1: Programmed cell death ligand 1
*P.*: *Plasmodium*
*S*. * japonicum*: *Schistosoma* *japonicum*
*S*. *mansoni*: *Schistosoma* *mansoni*
Spp.: Species
*T. solium*: *Taenia solium*
Treg: Tregulatory
*T*. *cruzi*: *Trypanosoma* *cruzi*
TME: Tumour microenvironment
*T*. * gondii*: *Toxoplasma* *gondii*
WHO: World Health Organization

## References

[1] World Health Organization. worldwide cancer statistics. Geneva: World Health Organization; 2024.

[2] Kaur R, Bhardwaj A, Gupta S. Cancer treatment therapies: traditional to modern approaches to combat cancers. Mol Biol Rep. 2023;50(11):9663–76. 10.1007/s11033-023-08809-3. 37828275 10.1007/s11033-023-08809-3

[3] Ramos A, Sadeghi S, Tabatabaeian H. Battling chemoresistance in cancer: root causes and strategies to uproot them. Int J Mol Sci. 2021. 10.3390/ijms22179451. 34502361 10.3390/ijms22179451 PMC8430957

[4] World Health Organization. Cancer Fact sheet. Geneva: World Health Organization; 2023.

[5] Rui R, Zhou L, He S. Cancer immunotherapies: advances and bottlenecks. Front Immunol. 2023;14:1212476–93. 10.3389/fimmu.2023.1212476. 37691932 10.3389/fimmu.2023.1212476 PMC10484345

[6] Feola S, Chiaro J, Martins B, Cerullo V. Uncovering the tumor antigen landscape: what to know about the discovery process. Cancers. 2020;12(6):1660–88. 10.3390/cancers12061660. 32585818 10.3390/cancers12061660 PMC7352969

[7] Smallwood TB, Giacomin PR, Loukas A, Mulvenna JP, Clark RJ, Miles JJ. Helminth immunomodulation in autoimmune disease. Front Immunol. 2017;8:453–68. 10.3389/fimmu.2017.00453. 28484453 10.3389/fimmu.2017.00453 PMC5401880

[8] Wolday D, Gebrecherkos T, Arefaine ZG, Kiros YK, Gebreegzabher A, Tasew G, et al. Effect of co-infection with intestinal parasites on COVID-19 severity: a prospective observational cohort study. EClinicalMedicine. 2021;39:101054–63. 10.1016/j.eclinm.2021.101054. 34368662 10.1016/j.eclinm.2021.101054 PMC8324426

[9] Gürel T, Umur Ş. Can parasites be useful? Turkiye Parazitol Derg. 2024;48(2):120–7. 10.4274/tpd.galenos.2024.43760. 38958490 10.4274/tpd.galenos.2024.43760

[10] Eissa MM, Allam SR, el Azzouni MZ, Baddour NM. Autoclaved cercarial vaccine: a new hope against schistosomiasis parasitologic, histopathologic and immunologic studies. J Egypt Soc Parasitol. 1998;28(2):461–79. 9707675

[11] Eissa MM, El-Azzouni MZ, Baddour NM, Boulos LM. Vaccination trial against experimental trichrinellosis using autoclaved *Trichinella**spiralis* larvae vaccine (ATSLV). J Egypt Soc Parasitol. 2003;33(1):219–28. 12739813

[12] Eissa MM, El-Azzouni MZ, Mady RF, Fathy FM, Baddour NM. Initial characterization of an autoclaved *Toxoplasma* vaccine in mice. Exp Parasitol. 2012;131(3):310–6. 10.1016/j.exppara.2012.05.001. 22595548 10.1016/j.exppara.2012.05.001

[13] Tosyali OA, Allahverdiyev A, Bagirova M, Abamor ES, Aydogdu M, Dinparvar S, et al. Nano-co-delivery of lipophosphoglycan with soluble and autoclaved *Leishmania* antigens into PLGA nanoparticles: evaluation of *in vitro* and *in**vivo* immunostimulatory effects against visceral leishmaniasis. Mater Sci Eng C Mater Biol Appl. 2021;120:111684–96. 10.1016/j.msec.2020.111684. 33545846 10.1016/j.msec.2020.111684

[14] Eissa MM, Allam SR, El-Azzouni MZ, Maged HR, Dessouky IS. Further studies on autoclaved cercarial vaccine against schistosomiasis: safety, longevity and stability. J Egypt Soc Parasitol. 2003;33(2):541–60. 14964666

[15] Eissa MM, Mostafa DK, Ghazy AA, El Azzouni MZ, Boulos LM, Younis LK. Anti-arthritic activity of *Schistosoma**mansoni* and *Trichinella**spiralis* derived-antigens in adjuvant arthritis in rats: role of FOXP3^+^ Treg Cells. PLoS ONE. 2016;11(11):165916–36. 10.1371/journal.pone.0165916. 10.1371/journal.pone.0165916 PMC5089557 27802332

[16] El Skhawy N, Eissa MM, Allam M, Eleryan EM. Immunomodulatory role of *Trichinella**spiralis*-derived antigen on imiquimod-induced psoriasis in mice model. Parasitol Res. 2024;123(11):397–409. 10.1007/s00436-024-08415-7. 39592463 10.1007/s00436-024-08415-7

[17] Eissa MM, Ismail CA, El-Azzouni MZ, Ghazy AA, Hadi MA. Immuno-therapeutic potential of *Schistosoma**mansoni* and *Trichinella**spiralis* antigens in a murine model of colon cancer. Invest New Drugs. 2019;37(1):47–56. 10.1007/s10637-018-0609-6. 29808307 10.1007/s10637-018-0609-6

[18] Eissa MM, Gaafar MR, Younis LK, Ismail CA, El Skhawy N. Prophylactic antineoplastic activity of *Toxoplasma**gondii* RH derived antigen against Ehrlich solid carcinoma with evidence of shared antigens by comparative immunoblotting. Infect Agent Cancer. 2023;18(1):21–34. 10.1186/s13027-023-00500-3. 37029378 10.1186/s13027-023-00500-3 PMC10082516

[19] Ismail CA, Eissa MM, Gaafar MR, Younis LK, El Skhawy N. *Toxoplasma**gondii*-derived antigen modifies tumor microenvironment of Ehrlich solid carcinoma murine model and enhances immunotherapeutic activity of cyclophosphamide. Med Oncol. 2023;40(5):136–49. 10.1007/s12032-023-01994-y. 37014499 10.1007/s12032-023-01994-y PMC10073061

[20] Lu J, Wei N, Zhu S, Chen X, Gong H, Mi R, et al. Exosomes derived from dendritic cells infected with *Toxoplasma**gondii* show antitumoral activity in a mouse model of colorectal cancer. Front Oncol. 2022;12: 899737. 10.3389/fonc.2022.899737. 35600363 10.3389/fonc.2022.899737 PMC9114749

[21] Daneshpour S, Kefayat AH, Mofid MR, Rostami Rad S, Yousofi DH. Effect of hydatid cyst fluid antigens on induction of apoptosis on breast cancer cells. Adv Biomed Res. 2019;8:27–35. 10.4103/abr.abr_220_18. 31123670 10.4103/abr.abr_220_18 PMC6477833

[22] Jafari H, Mahami-Oskouei M, Spotin A, Baradaran B, Shanehbandi D, Baghbanzadeh A, et al. Microrna-1 inhibits the growth of breast cancer cells MBA-MB-231 and MCF-7 treated with hydatid cyst fluid. J Trop Med. 2024;2024:7474039. 10.1155/2024/7474039. 38504949 10.1155/2024/7474039 PMC10950417

[23] Ranasinghe SL, Boyle GM, Fischer K, Potriquet J, Mulvenna JP, McManus DP. Kunitz type protease inhibitor EgKI-1 from the canine tapeworm *Echinococcus**granulosus* as a promising therapeutic against breast cancer. PLoS ONE. 2018;13(8):200433–49. 10.1371/journal.pone.0200433. 10.1371/journal.pone.0200433 PMC6118354 30169534

[24] Altun A, Saraydin SU, Soylu S, Inan DS, Yasti C, Ozdenkaya Y, et al. Chemopreventive effects of hydatid disease on experimental breast cancer. Asian Pac J Cancer Prev. 2015;16(4):1391–5. 10.7314/apjcp.2015.16.4.1391. 25743804 10.7314/apjcp.2015.16.4.1391

[25] Shakibapour M, Shojaie B, Yousofi DH. Immunization with hydatid cyst wall antigens can inhibit breast cancer through changes in serum levels of Th1/Th2 cytokines. Int J Prev Med. 2020;11:189–96. 10.4103/ijpvm.IJPVM_311_19. 33815713 10.4103/ijpvm.IJPVM_311_19 PMC8000162

[26] Shakibapour M, Kefayat A, Reza Mofid M, Shojaie B, Mohamadi F, Maryam Sharafi S, et al. Anti-cancer immunoprotective effects of immunization with hydatid cyst wall antigens in a non-immunogenic and metastatic triple-negative murine mammary carcinoma model. Int Immunopharmacol. 2021;99:107955–65. 10.1016/j.intimp.2021.107955. 34247052 10.1016/j.intimp.2021.107955

[27] Schcolnik-Cabrera A, Juárez M, Oldak B, Cruz-Rivera M, Flisser A, Dueñas-González A, et al. *In**vitro* employment of recombinant *Taenia**solium* calreticulin as a novel strategy against breast and ovarian cancer stem-like cells. Arch Med Res. 2020;51(1):65–75. 10.1016/j.arcmed.2019.12.003. 32097797 10.1016/j.arcmed.2019.12.003

[28] Wang X, Huang X, Li H, Yang S, Wang G, Yuan H. Analysis of genes differentially expressed by MCF-7 cells challenged with *Trichinella**spiralis* larval antigen. Zhongguo bing yuan sheng wu xue za zhi. 2019;14(7):750–4.

[29] Pan J, Ma M, Qin L, Kang Z, Adah D, Tao Z, et al. *Plasmodium* infection inhibits triple negative 4T1 breast cancer potentially through induction of CD8^(+)^ T cell-mediated antitumor responses in mice. Biomed Pharmacother. 2021;138:111406–15. 10.1016/j.biopha.2021.111406. 33676307 10.1016/j.biopha.2021.111406

[30] Hibbs JB Jr, Lambert LH Jr, Remington JS. Resistance to murine tumors conferred by chronic infection with intracellular protozoa, *Toxoplasma**gondii* and *Besnoitia**jellisoni*. J Infect Dis. 1971;124(6):587–92. 10.1093/infdis/124.6.587. 5127071 10.1093/infdis/124.6.587

[31] Lv Q, Li J, Gong P, Xing S, Zhang X. *Neospora**caninum*: *In**vitro* culture of tachyzoites in MCF-7 human breast carcinoma cells. Exp Parasitol. 2010;126(4):536–9. 10.1016/j.exppara.2010.06.006. 20566363 10.1016/j.exppara.2010.06.006

[32] Caner A, Sadıqova A, Erdoğan A, Namlıses D, Nalbantsoy A, Oltulu F, et al. Targeting of antitumor ımmune responses with live-attenuated *Leishmania* strains in breast cancer model. Breast Cancer. 2020;27(6):1082–95. 10.1007/s12282-020-01112-0. 32472473 10.1007/s12282-020-01112-0

[33] Ubillos L, Freire T, Berriel E, Chiribao ML, Chiale C, Festari MF, et al. *Trypanosoma**cruzi* extracts elicit protective immune response against chemically induced colon and mammary cancers. Int J Cancer. 2016;138(7):1719–31. 10.1002/ijc.29910. 26519949 10.1002/ijc.29910

[34] Akgül H, Tez M, Unal AE, Keşkek M, Sayek I, Ozçelik T. *Echinococcus* against cancer: why not? Cancer. 2003;98(9):1999–2000. 10.1002/cncr.11752. 14584087 10.1002/cncr.11752

[35] Zaghloul MS, Zaghloul TM, Bishr MK, Baumann BC. Urinary schistosomiasis and the associated bladder cancer: update. J Egypt Natl Canc Inst. 2020;32(1):44–55. 10.1186/s43046-020-00055-z. 33252773 10.1186/s43046-020-00055-z PMC13317092

[36] Von Bülow V, Lichtenberger J, Grevelding CG, Falcone FH, Roeb E, Roderfeld M. Does *Schistosoma**mansoni* facilitate carcinogenesis? Cells. 2021;10(8):1982–99. 10.3390/cells10081982. 34440754 10.3390/cells10081982 PMC8393187

[37] Sheng S, Chen B, Xu R, Han Y, Mao D, Chen Y, et al. A prognostic model for *Schistosoma**japonicum* infection-associated liver hepatocellular carcinoma: strengthening the connection through initial biological experiments. Infect Agent Cancer. 2024;19(1):10–29. 10.1186/s13027-024-00569-4. 38515119 10.1186/s13027-024-00569-4 PMC10956344

[38] Acharya S, Da’dara AA, Skelly PJ. Schistosome immunomodulators. PLoS Pathog. 2021;17(12): e1010064. 10.1371/journal.ppat.1010064. 34969052 10.1371/journal.ppat.1010064 PMC8718004

[39] Tang CL, Yu XH, Li Y, Zhang RH, Xie J, Liu ZM. *Schistosoma**japonicum* soluble egg antigen protects against type 2 diabetes in Lepr^db/db^ mice by enhancing regulatory T cells and TH2 cytokines. Front Immunol. 2019;10:1471. 10.3389/fimmu.2019.01471. 31297120 10.3389/fimmu.2019.01471 PMC6607994

[40] Li Z, Wang X, Zhang W, Yang W, Xu B, Hu W. Excretory/secretory products from *Schistosoma**japonicum* eggs alleviate ovalbumin-induced allergic airway inflammation. PLoS Negl Trop Dis. 2023;17(10): e0011625. 10.1371/journal.pntd.0011625. 37788409 10.1371/journal.pntd.0011625 PMC10547495

[41] Elliott DE, Li J, Blum A, Metwali A, Qadir K, Urban JF Jr, et al. Exposure to schistosome eggs protects mice from TNBS-induced colitis. Am J Physiol Gastrointest Liver Physiol. 2003;284(3):G385–91. 10.1152/ajpgi.00049.2002. 12431903 10.1152/ajpgi.00049.2002

[42] Hou X, Zhu F, Zheng W, Jacques ML, Huang J, Guan F, et al. Protective effect of *Schistosoma**japonicum* eggs on TNBS-induced colitis is associated with regulating Treg/Th17 balance and reprogramming glycolipid metabolism in mice. Front Cell Infect Microbiol. 2022;12:1028899. 10.3389/fcimb.2022.1028899. 36304936 10.3389/fcimb.2022.1028899 PMC9592807

[43] Pereira FE, Raso P, Coelho PM. Evolution of sarcoma 180 (ascitic tumor) in mice infected with *Schistosoma**mansoni*. Rev Soc Bras Med Trop. 1986;19(1):39–42. 10.1590/s0037-86821986000100009. 3120251 10.1590/s0037-86821986000100009

[44] Yang F, Sun X, Shen J, Yu LP, Liang JY, Zheng HQ, et al. A recombined protein (rSj16) derived from *Schistosoma**japonicum* induces cell cycle arrest and apoptosis of murine myeloid leukemia cells. Parasitol Res. 2013;112(3):1261–72. 10.1007/s00436-012-3260-8. 23319265 10.1007/s00436-012-3260-8

[45] Hu C, Zhu S, Wang J, Lin Y, Ma L, Zhu L, et al. *Schistosoma**japonicum* miRNA-7-5p inhibits the growth and migration of hepatoma cells via cross-species regulation of S-phase kinase-associated protein 2. Front Oncol. 2019;9:175. 10.3389/fonc.2019.00175. 30967999 10.3389/fonc.2019.00175 PMC6443022

[46] Pekkle HY, Liang TR, Jiang SJ, Peng SY. *Schistosoma**mansoni* soluble egg antigen suppresses colorectal cancer growth *in vitro* and *in**vivo*. J Microbiol Immunol Infect. 2024. 10.1016/j.jmii.2024.11.009. 10.1016/j.jmii.2024.11.009 39653602

[47] Yao X, Cao Y, Lu L, Xu Y, Chen H, Liu C, et al. *Plasmodium* infection suppresses colon cancer growth by inhibiting proliferation and promoting apoptosis associated with disrupting mitochondrial biogenesis and mitophagy in mice. Parasit Vectors. 2022;15(1):192–204. 10.1186/s13071-022-05291-x. 35668501 10.1186/s13071-022-05291-x PMC9169289

[48] Lin Y, Zhu S, Hu C, Wang J, Jiang P, Zhu L, et al. Cross-species suppression of hepatoma cell growth and migration by a *Schistosoma**japonicum* microRNA. Mol Ther Nucleic Acids. 2019;18:400–12. 10.1016/j.omtn.2019.09.006. 31655260 10.1016/j.omtn.2019.09.006 PMC6831938

[49] Adah D, Yang Y, Liu Q, Gadidasu K, Tao Z, Yu S, et al. *Plasmodium* infection inhibits the expansion and activation of MDSCs and Tregs in the tumor microenvironment in a murine Lewis lung cancer model. Cell Commun Signal. 2019;17(1):32–44. 10.1186/s12964-019-0342-6. 30979375 10.1186/s12964-019-0342-6 PMC6461823

[50] Loures MA, Andrade A, Peralta JM. Local response in mouse tumor treated with *Schistosoma**mansoni* antigen. Mem Inst Oswaldo Cruz. 1991;86(3):127–8. 10.1590/s0074-02761991000700027. 1668995 10.1590/s0074-02761991000700027

[51] Vafae EA, Ghaffarifar F, Muhammad HZ, Dalimi A. Anticancer activity of hydatid cyst fluid along with antigen B on tumors induced by 4t1 breast cancer cell in a BALB/c mice model. Iran J Parasitol. 2022;17(2):240–9. 10.18502/ijpa.v17i2.9542. 36032740 10.18502/ijpa.v17i2.9542 PMC9363258

[52] Silva J, Faustino-Rocha AI, Duarte JA, Oliveira PA. Realistic aspects behind the application of the rat model of chemically-induced mammary cancer: practical guidelines to obtain the best results. Vet World. 2023;16(6):1222–30. 10.14202/vetworld.2023.1222-1230. 37577198 10.14202/vetworld.2023.1222-1230 PMC10421542

[53] Weidner N, Semple JP, Welch WR, Folkman J. Tumor angiogenesis and metastasis correlation in invasive breast carcinoma. N Engl J Med. 1991;324(1):1–8. 10.1056/nejm199101033240101. 1701519 10.1056/NEJM199101033240101

[54] Han J, Gu X, Li Y, Wu Q. Mechanisms of BCG in the treatment of bladder cancer-current understanding and the prospect. Biomed Pharmacother. 2020;129: 110393. 10.1016/j.biopha.2020.110393. 32559616 10.1016/j.biopha.2020.110393

[55] Kidner TB, Morton DL, Lee DJ, Hoban M, Foshag LJ, Turner RR, et al. Combined intralesional Bacille Calmette-Guérin (BCG) and topical imiquimod for in-transit melanoma. J Immunother. 2012;35(9):716–20. 10.1097/CJI.0b013e31827457bd. 23090081 10.1097/CJI.0b013e31827457bd PMC3674843

[56] Ferrucci PF, Pala L, Conforti F, Cocorocchio E. Talimogene laherparepvec (T-vec): an intralesional cancer immunotherapy for advanced melanoma. Cancers. 2021;13(6):1383–97. 10.3390/cancers13061383. 33803762 10.3390/cancers13061383 PMC8003308

[57] Barra F, Leone Roberti Maggiore U, Bogani G, Ditto A, Signorelli M, Martinelli F, et al. New prophylactics human papilloma virus (HPV) vaccines against cervical cancer. J Obstet Gynaecol. 2019;39(1):1–10. 10.1080/01443615.2018.1493441. 30370796 10.1080/01443615.2018.1493441

[58] Dietz CA, Wedemeyer H. Vaccination against hepatitis B as prevention for hepatocellular carcinoma. Onkologe (Berl). 2022;28(1):15–22. 10.1007/s00761-021-01036-0. 34658542 10.1007/s00761-021-01036-0 PMC8511853

[59] Eissa MM, Salem AE, El Skhawy N. Parasites revive hope for cancer therapy. Eur J Med Res. 2024;29(1):489–545. 10.1186/s40001-024-02057-2. 39367471 10.1186/s40001-024-02057-2 PMC11453045

[60] Dasari S, Tchounwou PB. Cisplatin in cancer therapy: molecular mechanisms of action. Eur J Pharmacol. 2014;740:364–78. 10.1016/j.ejphar.2014.07.025. 25058905 10.1016/j.ejphar.2014.07.025 PMC4146684

[61] Jiang P, Wang J, Zhu S, Hu C, Lin Y, Pan W. Identification of a *Schistosoma**japonicum* MicroRNA that suppresses hepatoma cell growth and migration by targeting host FZD4 gene. Front Cell Infect Microbiol. 2022;12:786543–54. 10.3389/fcimb.2022.786543. 35174106 10.3389/fcimb.2022.786543 PMC8842725

[62] Hu C, Li Y, Pan D, Wang J, Zhu L, Lin Y, et al. A *Schistosoma**japonicum* microRNA exerts antitumor effects through inhibition of both cell migration and angiogenesis by targeting PGAM1. Front Oncol. 2021;11:652395–406. 10.3389/fonc.2021.652395. 34221971 10.3389/fonc.2021.652395 PMC8242254

[63] Salanti A, Clausen TM, Agerbæk M, Al Nakouzi N, Dahlbäck M, Oo HZ, et al. Targeting human cancer by a glycosaminoglycan binding malaria protein. Cancer Cell. 2015;28(4):500–14. 10.1016/j.ccell.2015.09.003. 26461094 10.1016/j.ccell.2015.09.003 PMC4790448

[64] Sheklakova LA, Kallinikova VD, Karpenko LP. Genetic heterogeneity of *Trypanosoma**cruzi* and its direct anticancer effect in cultured human tumor cells. Bull Exp Biol Med. 2003;135(1):89–92. 10.1023/a:1023466517225. 12717523 10.1023/a:1023466517225

[65] Borges BC, Uehara IA, Dos Santos MA, Martins FA, de Souza FC, Junior ÁF, et al. The recombinant protein based on *Trypanosoma**cruzi* P21 interacts with CXCR4 receptor and abrogates the invasive phenotype of human breast cancer cells. Front Cell Dev Biol. 2020;8:569729–41. 10.3389/fcell.2020.569729. 33195200 10.3389/fcell.2020.569729 PMC7604327

[66] Wu X, Sun L, Zhang L, Liu ZQ, Luo Q, Zhang LX. Impact of *Toxoplasma**gondii* on the proliferation and apoptosis of tumor cell lines. Zhongguo Ji Sheng Chong Xue Yu Ji Sheng Chong Bing Za Zhi. 2012;30(2):157–9. 22908820

[67] Eissa MM, Gaafar MR, Younis LK, Ismail CA, El Skhawy N. Evaluation of cytotoxic activity of live *Toxoplasma**gondii* tachyzoites and toxoplasma antigen on MCF-7 human breast cancer cell line. EUREKA Life Sci. 2022;2:45–50. 10.21303/2504-5695.2022.002409.

[68] Ye H, Zhou X, Zhu B, Xiong T, Huang W, He F, et al. *Toxoplasma**gondii* suppresses proliferation and migration of breast cancer cells by regulating their transcriptome. Cancer Cell Int. 2024;24(1):144–58. 10.1186/s12935-024-03333-1. 38654350 10.1186/s12935-024-03333-1 PMC11040860

[69] Atalay-Şahar E, Döşkaya M, Karakavuk M, Can H, Gül A, Gürüz AY, et al. *Toxoplasma**gondii* destroys Her2/Neu-expressing mammary cancer cells *in**vitro* using a continuous feed medium approach. J Infect Dev Ctries. 2020;14(10):1204–9. 10.3855/jidc.12820. 33175718 10.3855/jidc.12820

[70] Azevedo AC, Cristina OR, Rodrigues CC, Teixeira SC, Borges BC, Vieira SC. *Trypanosoma**cruzi* infection induces proliferation and impairs migration of a human breast cancer cell line. Exp Parasitol. 2023;245:108443–9. 10.1016/j.exppara.2022.108443. 36526003 10.1016/j.exppara.2022.108443

[71] Huang M, Xia Y, Li K, Shao F, Feng Z, Li T, et al. Carcinogen exposure enhances cancer immunogenicity by blocking the development of an immunosuppressive tumor microenvironment. J Clin Invest. 2023;133(20):166494–509. 10.1172/jci166494. 10.1172/JCI166494 PMC10575722 37843274

[72] Leser C, Dorffner G, Marhold M, Rutter A, Döger M, Singer C, et al. Liver function indicators in patients with breast cancer before and after detection of hepatic metastases-a retrospective study. PLoS ONE. 2023;18(3):278454–64. 10.1371/journal.pone.0278454. 10.1371/journal.pone.0278454 PMC9983906 36867604

[73] Akhouri V, Kumari M, Kumar A. Therapeutic effect of *Aegle**marmelos* fruit extract against DMBA induced breast cancer in rats. Sci Rep. 2020;10(1):18016–28. 10.1038/s41598-020-72935-2. 33093498 10.1038/s41598-020-72935-2 PMC7581526

[74] Weatherly NF. Increased survival of Swiss mice given sublethal infections of *Trichinella**spiralis*. J Parasitol. 1970;56(4):748–52. 5460510

[75] Apanasevich VI, Britov VA, Zban’ IuV. Antitumor cross-resistance of trichinosis. Vopr Onkol. 2002;48(2):223–6. 12227073

[76] Xu LQ, Yao LJ, Jiang D, Zhou LJ, Chen M, Liao WZ, et al. A uracil auxotroph *Toxoplasma**gondii* exerting immunomodulation to inhibit breast cancer growth and metastasis. Parasit Vectors. 2021;14(1):601–14. 10.1186/s13071-021-05032-6. 34895326 10.1186/s13071-021-05032-6 PMC8665513

[77] Abello-Cáceres P, Pizarro-Bauerle J, Rosas C, Maldonado I, Aguilar-Guzmán L, González C, et al. Does native *Trypanosoma**cruzi* calreticulin mediate growth inhibition of a mammary tumor during infection? BMC Cancer. 2016;16(1):731–43. 10.1186/s12885-016-2764-5. 27619675 10.1186/s12885-016-2764-5 PMC5020520

[78] Amaravadi RK, Thompson CB. The roles of therapy-induced autophagy and necrosis in cancer treatment. Clin Cancer Res. 2007;13(24):7271–9. 10.1158/1078-0432.Ccr-07-1595. 18094407 10.1158/1078-0432.CCR-07-1595

[79] Ding JH, Xiao Y, Zhao S, Xu Y, Xiao YL, Shao ZM, et al. Integrated analysis reveals the molecular features of fibrosis in triple-negative breast cancer. Mol Ther Oncolytics. 2022;24:624–35. 10.1016/j.omto.2022.02.003. 35284626 10.1016/j.omto.2022.02.003 PMC8898759

[80] Zheng J, Hao H. The importance of cancer-associated fibroblasts in targeted therapies and drug resistance in breast cancer. Front Oncol. 2023;13:1333839–58. 10.3389/fonc.2023.1333839. 38273859 10.3389/fonc.2023.1333839 PMC10810416

[81] Li JJ, Tsang JY, Tse GM. Tumor microenvironment in breast cancer-updates on therapeutic implications and pathologic assessment. Cancers. 2021;13(16):4233–60. 10.3390/cancers13164233. 34439387 10.3390/cancers13164233 PMC8394502

[82] Chidananda MG. Ki-67 index and its correlation with clinical and pathological variables in breast cancer. Indian J Surg Oncol. 2023;14(4):943–8. 10.1007/s13193-023-01833-6. 38187860 10.1007/s13193-023-01833-6 PMC10766571

[83] Jääskeläinen MM, Tiainen S, Siiskonen H, Ahtiainen M, Kuopio T, Rönkä A, et al. The prognostic and predictive role of tumor-infiltrating lymphocytes (FoxP3 + and CD8 +) and tumor-associated macrophages in early HER2 + breast cancer. Breast Cancer Res Treat. 2023;201(2):183–92. 10.1007/s10549-023-07017-8. 37428418 10.1007/s10549-023-07017-8 PMC10361875

[84] Saastad SA, Skjervold AH, Ytterhus B, Engstrøm MJ, Bofin AM. PD-L1 protein expression in breast cancer. J Clin Pathol. 2023;11:730–6. 10.1136/jcp-2023-208942. 10.1136/jcp-2023-208942 37553245

[85] Goyal AV, Shukla S, Acharya S, Vagha S, Jajoo S. Correlation of microvessel density with histopathological parameters of carcinoma breast. Indian J Med Res. 2023;158(4):417–22. 10.4103/ijmr.ijmr_1588_22. 38006344 10.4103/ijmr.ijmr_1588_22 PMC10793828

[86] Marelli-Berg FM, Clement M, Mauro C, Caligiuri G. An immunologist’s guide to CD31 function in T-cells. J Cell Sci. 2013;126(11):2343–52. 10.1242/jcs.124099. 23761922 10.1242/jcs.124099

[87] López NC, Valck C, Ramírez G, Rodríguez M, Ribeiro C, Orellana J, et al. Antiangiogenic and antitumor effects of *Trypanosoma**cruzi* Calreticulin. PLoS Negl Trop Dis. 2010;4(7):730–9. 10.1371/journal.pntd.0000730. 10.1371/journal.pntd.0000730 PMC2897838 20625551

[88] Deng X, Zheng H, Zhou D, Liu Q, Ding Y, Xu W, et al. Antitumor effect of intravenous immunization with malaria genetically attenuated sporozoites through induction of innate and adaptive immunity. Int J Clin Exp Pathol. 2016;9(2):978–86.

[89] Kim JO, Jung SS, Kim SY, Kim TY, Shin DW, Lee JH, et al. Inhibition of Lewis lung carcinoma growth by *Toxoplasma**gondii* through induction of Th1 immune responses and inhibition of angiogenesis. J Korean Med Sci. 2007;22:38–46. 10.3346/jkms.2007.22.S.S38. 10.3346/jkms.2007.22.S.S38 PMC2694397 17923753

[90] Pyo KH, Jung BK, Chai JY, Shin EH. Suppressed CD31 expression in sarcoma-180 tumors after injection with *Toxoplasma**gondii* lysate antigen in BALB/c mice. Korean J Parasitol. 2010;48(2):171–4. 10.3347/kjp.2010.48.2.171. 20585536 10.3347/kjp.2010.48.2.171 PMC2892575

[91] Hunter CA, Yu D, Gee M, Ngo CV, Sevignani C, Goldschmidt M, et al. Cutting edge: systemic inhibition of angiogenesis underlies resistance to tumors during acute toxoplasmosis. J Immunol. 2001;166(10):5878–81. 10.4049/jimmunol.166.10.5878. 11342601 10.4049/jimmunol.166.10.5878

